# Abstract of the Proceedings of the Buffalo Medical Association

**Published:** 1862-02

**Authors:** Julius F. Miner

**Affiliations:** Secretary


### ART, II.—*Abstract of the Proceedings of the Buffalo Medical Association.*

TUESDAY EVENING, January 7, 1862,

The Association met at the usual hour.

Present—Dr. C. C. F. Gay, President in the Chair; Drs. Samo, White, Congar, Wyckoff, Eastman, Washburn, Kempson, Strong, Gould and Miner.

Minutes of last meeting were read and approved.

On motion of *Dr. White,* voted that Dr. Charles Washburn, of Fredonia, be invited to participate in the transactions of the society.

Report of the Committee to audit Treasurer's account, was received, adopted, and the Committee discharged.

*Dr Rochester*, remarked, that he had some time since, promised to report to the Association, the result of the injection of a solution of persulphate of iron into a vascular tumor. He was consulted on the first of August 1861, by a lady from the country, who informed him that she was thrown from a carriage in 1857, and received an injury on the back of the head which confined her for some weeks to her bed, that about six weeks after the accident she discovered a slight swelling at the seat of the injury to which she paid little attention, until she wounded it combing her hair, causing a hemorrhage so profuse that medical aid was summoned. The tumor was pronounced aneurismal and cold, stypties and pressure failing to control the bleeding, the occipital artery was taken up, and pressure with a metalic disc continued, These measures arrested the hemorrhage and for some months the growth of the tumor. About this time she consulted Dr. Willard Parker of New York, who advised her to wear the metallic plate and not to undergo another operation. The tumor however continued to increase, yet so slowly that it was only perceptible on examination after long intervals. In June 1861, it was again accidently wounded with a comb, and another severe hemorrhage ensued, but which pressure sufficed to arrest. On examination, the tumor was found a little above the occipital protuberance, it was about an inch and a half in diameter, and, as estimated, three quarter of an inch in thickness, it had an elastic feeling and pulsated strongly especially on its lower and left margin. The scalp was not discolored. I was regarded as a traumatic aneurism, and it was proposed to inject it with persulphate of iron. To this the patient assented, and a hypodermic syringe was half filled with equal parts of Squibb's solution of persulphate of iron and water; The needle was passed into the centre of the tumor and its point was found to have great freedom of motion. It was moved about freely, as advised by Gross, to scarify and break up the minute vessels of the lining membrane of the sac, and the syringe was then discharged of all its contents. The patient immediately complained of very severe pain in the tumor and in a *portion* of the *forehead*, the seat of the pain in the latter corresponding with a *vein*, now very prominent and apparent, but not before observed.— The needle was withdrawn and no blood escaped except a few drops from the integument. The tumor instantly became hard and swollen and the skin over it red and tense. Pulsation could no longer be detected. The pain continued so severe that after some hours, it required a full dose of morphine to allay it. At the end of a week, the tumor remained hard and the swelling had subsided, slight pulsation could however be detected at several points. The operation was repeated as before, except that the solution of iron was undiluted. Very severe pain again succeeded the injection, particularly in the forehead, the vein before mentioned becoming prominent black and hard. At the expiration of the second week, the tumor was hard, contracted, painless and pulseless. On the twentieth day after the first in_ jection and the twelfth after all pulsation had ceased, the tumor was laid open with a scalpel, with the expectation of removing a hard clot. This however did not appear, but in its place, spouted profuse arterial hemorrhage. A free crucial incision down to the bone was instantly made, into which was inserted a sponge saturated with solution of persulphate of iron, over this a firm compress was placed; and the whole secured by a very tight bandage. This however was not accomplished before a good deal of blood was lost, notwithstanding firm digital compression faithfully and skillfully maintained by Mr. H. P. Babcock. No more hemorrhage ensued after the adjustment of the compress. This was removed on the 26th, of August. The sponge was seen hard dry and black and so firmly imbedded in the incision that it was not deemed proper to attempt its removal. The lady returned to the country on the first of September. In November a letter was received from her in which she stated that the sponge remained in situ for nearly a month, it then came away and the tumor had disappeared. Dr. Rochester remarked that before opening the tumor he had wished to make one or two more injections, but the patient was very anxious to go home, and from the long and entire cessation of pulsation he certainly supposed it was safe to make the incision which revealed the failure of the hypodermic treatment. He was at a loss to account for the swelling and induration of the vein on the forehead, it looked as if the tumor migh been erectile instead of aneurismal and to have had venous as well as arterial connections.

*Dr Washburn* related a case where measles supervened upon diphtheria The exudation was very abundant, covering the fauces and uvula, standing out prominently for about one week: during this time measles made its appearance in well marked form, and the two diseases continued together until about the eighth day when the child died in convulsions, Dr. W. would inquire, if in the experience of the physicians present, it was common for measles to supervene upon, or appear simultaneously with diphtheria?

*Dr. Rochester* thought that in an epidemic of measles such cases would be liable to occur, the reverse being very frequently observed. Diphtheria supervening upon, and complicating measles.

*Dr. Eastman* had treated recently a patient whose family had all previously been sick with scarlet fever. The father was soon after attacked by the disease, and when the eruption began to fade, diphtheria made its appearance, and in a few days erysipelas. Under this complication of maladies the patient sank exhausted. Any one of these diseases, single handed, being enough to be borne.

*Dr. Congar* coincided with the opinions expressed and related a case under his care in which erysipelas first appeared upon the nose; soon the throat commenced to swell and the diphtheritic exudation was very distinct, This patient passed through the diseases and convalesed very rapidly.

*Dr. Wyckoff* related cases of mumps and diphtheria appearing simultaneously or the swelling of mumps would seem first to appear, when afterwards diphtheritic exudation would become distinct and extensive. Expressed some doubt as to the character of the swelling, thinking possibly the glandular enlargement might be in some cases a characteristic of diphtheria.

*Dr. Kempson* remarked that Dr. Miner had recently "invaded Canada," and advised in an interesting case which he should be very much gratified to hear described. Hoped nothing from America would ever invade the Queen's dominions more objectionable than her Surgeons, when the people and government would submit without war,

*Dr. Miner* remarked that he had visited a lady in Fort Erie, with a young physician of that place. Did not often report cases under the care of other physicians, but knew of no reason why a brief description could be in any way objectionable. Found the patient with rheumatic synovitis of the knee joint or what appeared to be such disease, though when visited by him it had lost the earlier rheumatic characteristics and wa ynovitis of the knee joint with effusion not only into the synovial cavity but also into the cellular tissue above and below the joint. The leg was flexed upon the thigh; there was great pain and tenderness on pressure or motion. At his second visit much the same conditions were present as at the first; twenty-four hours preceding, there had been most violent and protracted chill. Pulse rapid; skin at times hot and again bathed in cold perspiration; fluctuation distinct with great swelling and redness. Present all the general symptoms and local appearances of purulent accumulation in the knee joint. Puncture was made with lancet in front, just below the patella, passing deeply through the greatly distended tissues towards the head of tibia. Contrary to our expectation only two or three ounces of serum escaped and so far as observed no purulent deposit had taken place.

At his last visit the general appearances of the knee had greatly improved. Swelling, redness, tenderness and pain had abated, and the patient was comparatively very comfortable. The leg was laid upon a splint to prevent contraction and motion. The pain had been intense, and motion impossible, but as last seen there was every indication of a pleasant recovery.

*Dr. Rochester* said that the case refered to by Dr, Kempson and reported by Dr. Miner, reminded him of one he had visited more than a year ago in consultation with Dr. Congar. The patient, a middle aged man, presented all the general and local symptoms of synovitis with purulent deposits in and around the joint, the thigh and leg both being greatly swollen and giving to the touch the idea of fluctuation from pus. An exploring needle was passed deep into the tissues in several places, but no pus was found.—Tonics, bandages and splints were employed, and the man made a good re_covery. Dr. Rochester was not averse to making a free incision into the joint, if satisfied of the presence of pus, but he would inquire of Dr. Miner' whether, if an exploring needle had been used by him, he would have made the free incision (into the joint?) from which the two ounces of serum were immediately discharged.

*Dr Miner* replied that he did not think the cavity of the joint was opened, though had its walls been distended as much as they appeared to be, the incision would unquestionably have reached that cavity. The discharge of serum was gradual and from the cellular tissue around the joint and was doubtless as much in quantity as had been estimated, perhaps much greater. Upon the point of opening the cavity of the joint had an ex ploring needle shown that only serum was effused he did not wish to give an unhesitating and unqualified opinion, to be applied indiscriminately to similar cases. The great fear of opening into a joint which had formerly prevailed among surgeons, had in great degree passed away, and when pus had accumulated there could be no difference of opinion, the propriety and necessity would be conceded by all. If the cavity of the knee joint was greatly distended with serum, and had remained so for some time, showing that absorption was inadquate for its ready removal, and pain was present in great degree, puncture with lancet, exploring trocar, or other instrument whereby the distention would be relieved, would in his opinion be the most proper and judicious treatment, most likely to relieve pain, and best calculated to prevent the formation of pus. While he did not care to defend the treatment adopted in the case referred to, and was willing, to say that had he not expected to find pus he might not at that time have advised the puncture, (not free opening) which was made, yet all the results of the case have most fully and unmistakably justified the treatment. The old idea that the admission of air into the cavity of a joint must almost certainly be productive of the most disasterous results has gradually given place to the view that it is not, after all, so much to be feared, and even some European surgeons of great reputation, have gone so far as to not only puncture, but also to inject Tinc. Iodine, with the view of changing the character of the effusion. In regard to the instrument used he wished to say one word. The lancet will allow the escape of the serum contained in the cellular tissue and is thus productive of good, at least in some cases, and might often prevent the small superficial abcesses which are apt to form, while all desired information is readily obtained by it. An exploring needle will with difficulty show even the character of the effusion, very little if anything escaping by its side or after its withdrawal, and inflamation, erysipelas or other accident is fully as liable to follow "its deep introduction in several places," as it is to succeed the judicious use of the lancet.

*Dr. White* gave an account of a medico legal case in which he had recently been called to give testimony as an expert. Mr Gibson, of Warsaw, Wyoming county, N. Y., was found with gun shot wound in the side which soon proved fatal. His life was very largely insured and the point was to show from the circumstances, nature of wound, &c., that death was accidental rather than intentional.

The various facts which proved the death accidental, apparently unimportant when seperately considered, were when connected, almost positive proof that the injury could not have been intentionally produced. Dr. Meacham of Warsaw, had promised to furnish a detailed account of the case for publication, consequently a more minute history would not be given at present.

*Dr. Eastman* had been consulted in the case. Regarded it an important one, and thought the profession would be greatly interested in it in a medico legal point of view.

*Voted* on motion of *Dr. Strong*, that Mr. E. S. Rich be permitted to remove the portrait of Dr. Trowbridge to the rooms of the Art Union exhibition.

*Dr. Gay* proposed Dr. Leon F. Harvey, for membership.

*Voted* to adjourn to the first Tuesday in February, 7½ o'clock P. M.

JULIUS F. MINER, Secretary.

